# Preclinical comparison of [^177^Lu]Lu-rhPSMA-10.1 and [^177^Lu]Lu-rhPSMA-10.2 for endoradiotherapy of prostate cancer: biodistribution and dosimetry studies

**DOI:** 10.1186/s41181-024-00246-2

**Published:** 2024-02-26

**Authors:** Alexander Wurzer, Francesco De Rose, Sebastian Fischer, Markus Schwaiger, Wolfgang Weber, Stephan Nekolla, Hans-Jürgen Wester, Matthias Eiber, Calogero D’Alessandria

**Affiliations:** 1 Department of Nuclear Medicine, University Hospital rechts der Isar, Technical University of Munich, Ismaninger Straße 22, 81675 Munich, Germany; 2 Chair of Pharmaceutical Radiochemistry, Technical University of Munich, Garching, Germany

**Keywords:** Prostate cancer, Radioligand therapy, Radiohybrid, rhPSMA, PSMA

## Abstract

**Background:**

Radiohybrid PSMA-targeted ligands (rhPSMA) have been introduced as a novel platform for theranostic applications. Among a variety of rhPSMA-ligands developed for radioligand therapy, two stereoisomers [^177^Lu]Lu-rhPSMA-10.1 and -10.2 have been synthesized and initially characterized in preclinical experiments with the aim to provide an optimized binding profile to human serum albumin, a reduction of charge, and thus accelerated kidney excretion, and unaffected or even improved tumor uptake. As both isomers showed similar in vitro characteristics and tumor uptake at 24 h post injection in tumor bearing mice and in order to identify the isomer with the most favorable pharmacokinetics for radioligand therapy, we carried out in-depth biodistribution and dosimetry studies in tumor-bearing and healthy mice.

**Results:**

rhPSMA-10.1 and -10.2 were radiolabeled with lutetium-177 according to the established procedures of other DOTA-based PSMA ligands and displayed a high and comparable stability in all buffers and human serum (> 97%, 24 h). Biodistribution studies revealed fast clearance from the blood pool (0.3–0.6%ID/g at 1 h) and other background tissues within 48 h. Distinctive differences were found in the kidneys, where [^177^Lu]Lu-rhPSMA-10.1 displayed lower initial uptake and faster excretion kinetics compared to [^177^Lu]Lu-rhPSMA-10.2 expressed by a 1.5-fold and ninefold lower uptake value at 1 h and 24 h in healthy animals, respectively. Tumor uptake was comparable and in the range of 8.6–11.6%ID/g for both isomers over 24 h and was maintained up to 168 h at a level of 2.2 ± 0.8 and 4.1 ± 1.4%ID/g for [^177^Lu]Lu-rhPSMA-10.1 and [^177^Lu]Lu-rhPSMA-10.2, respectively.

**Conclusion:**

Our preclinical data on biodistribution and dosimetry indicate a more favorable profile of [^177^Lu]Lu-rhPSMA-10.1 compared to [^177^Lu]Lu-rhPSMA-10.2 for PSMA-targeted radioligand therapy. [^177^Lu]Lu-rhPSMA-10.1 shows fast kidney clearance kinetics resulting in excellent tumor-to-organ ratios over a therapy relevant time course. Meanwhile, [^177^Lu]Lu-rhPSMA-10.1 is currently being investigated in clinical phase I/II studies in patients with mCRPC (NCT05413850), in patients with high-risk localized PC (NCT06066437, Nautilus Trial) and after external beam radiotherapy (NCT06105918).

**Supplementary Information:**

The online version contains supplementary material available at 10.1186/s41181-024-00246-2.

## Introduction

The FDA approval of [^177^Lu]Lu-PSMA-617 (Pluvicto™, PSMA: prostate-specific membrane antigen) in 2022 established a new treatment option for patients with metastatic castration resistant prostate cancer (mCRPC) (Fallah et al. 2023). In the VISION trial targeted radioligand therapy with [^177^Lu]Lu-PSMA-617 significantly improved overall survival compared with best supportive care (15.3 vs. 11.3 months) and radiographic progression-free survival (8.7 vs. 3.4 months) (Sartor et al. 2021). PSMA-I&T is another first-generation PSMA targeting ligand which can be labeled with lutetium-177 and is in advanced clinical development (NCT04647526, NCT05204927). In retrospective comparisons, pharmacokinetics and clinical efficacy of both agents is assumed to be comparable (Schuchardt et al. 2022; Kulkarni et al. 2016). Usually, biochemical response is achieved in 30–65% of patients, depending on patient selection criteria (Sartor et al. 2021; Heck et al. 2019; Rahbar et al. 2017).

Recently radiohybrid PSMA-targeted ligands (rhPSMA) have been introduced as a novel platform with potentially improved characteristics for theranostic applications (Wurzer et al. 2020a). rhPSMA radiopharmaceuticals combine a silicon–fluoride acceptor for easy and fast ^18^F-labeling and a chelator for complexation of a (radio)metal in one molecule. Respective ligand pairs of “^18^F/non-radioactive metal” and “^19^F/radiometal” are chemically identical and thus display identical pharmacokinetics, allowing their application for ^18^F-based PET-imaging and radiotherapy in a truly theranostic manner (Wurzer et al. 2020a, b). As a very first rh-ligand, [^18^F]Ga-rhPSMA-7.3 (POSLUMA®, flotufolastat F18, ^18^F-rhPSMA-7.3) was recently FDA-approved for imaging of patients with suspected prostate cancer recurrence (Jani et al. 2023; Surasi et al. 2023).

**Fig. 1:**
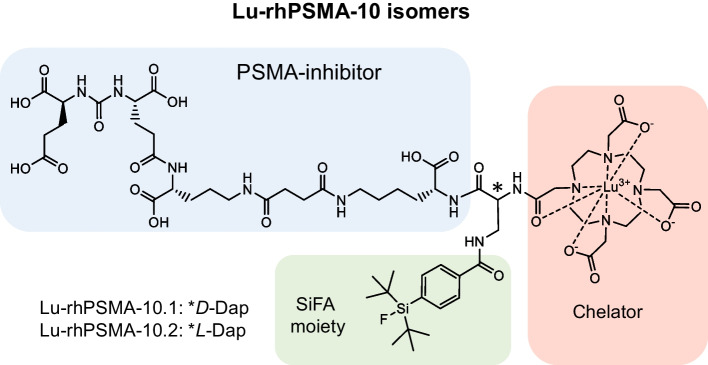
Chemical structure of the Lu-rhPSMA-10 isomers, differing in the stereoconfiguration of diaminopropionic acid (Dap) branching unit. *D*-Dap: rhPSMA-10.1 and *L*-Dap: rhPSMA-10.2. The PSMA inhibitor displays two moieties for radiolabeling: A silicon–fluoride acceptor (SiFA, colored in green), allowing ^18^F-labeling for PET, and a chelator (colored in orange) for complexation of radiometals

Preclinical analyses established [^177^Lu]Lu-rhPSMA-7.3 as a potential alternative to [^177^Lu]Lu-PSMA-I&T which has similar clearance kinetics and a similar radiation dose to healthy organs but superior tumor uptake and retention (Yusufi et al. 2021). In a small comparative clinical investigation, these differences were less pronounced (Feuerecker et al. 2021). With the aim of faster clearance from healthy organs, further investigations ultimately led to the development of the stereoisomeric pair, rhPSMA-10: rhPSMA-10.1 with a *D*-Dap (diaminopropionic acid) and rhPSMA-10.2 with *L*-Dap branching unit, respectively (Fig. 1). Both ligands provide, reduced overall charge and lower binding strength to human serum albumin (HSA) potentially enhancing clearance from healthy organs (Wurzer et al. 2022). After initial preclinical studies, rhPSMA-10.1 was prioritized for further development of an improved theranostic candidate (Wurzer et al. 2022).

In the present preclinical study, we investigate the effect of stereoisomerism on the pharmacokinetics of [^177^Lu]Lu-rhPSMA-10.1 and [^177^Lu]Lu-rhPSMA-10.2 in healthy and PSMA-positive LNCaP-tumor bearing mice in order to identify the rhPSMA-10 isomer with the most favorable characteristics for radioligand therapy.

## Materials and methods

### General information

rhPSMA-10.1 and rhPSMA-10.2 were obtained by Almac Sciences Scotland (Penicuik, UK). All reagents were purchased from Sigma-Aldrich (Merck Group, St. Louis, Missouri, United States). Phosphate buffered saline (PBS) pH 7.4 was prepared by the Hospital Pharmacy of the University Hospital rechts der Isar according to the following recipe: sodium chloride 66 mM, potassium chloride 1.3 mM, sodium hydrogenphospate dihydrate 4.0 mM, and potassium hydrogen phosphate 0.6 mM. The 0.9% NaCl solution was as well prepared by the Hospital Pharmacy. Human serum was obtained from PAN Biotech (Aidenbach, Regensburg, Germany).

### Cell culture

The human prostate cancer cell line LNCaP (DSMZ German Collection of Microorganisms and Cell Cultures GmbH, Braunschweig, Germany) was used for the establishment of tumor xenograft models. Cells were routinely screened for mycoplasma and authenticity was confirmed by short tandem repeat analysis. The cells were maintained as monolayer cultures in RPMI (Gibco, Carlsbad, US) containing 10% fetal bovine serum and Penicillin–Streptomycin (Gibco, Carlsbad, US) at 37 °C in a humidified CO_2_ atmosphere (5%).

### Animals and tumor xenograft model

Six-week old male CB17 SCID (severe combined immunodeficiency, Charles River Laboratories) mice were used for the animal experiments according to guidelines for the welfare and use of animals in cancer research experimentation. All experiments were approved by local authorities (animal license 55.2-1-54-2532-216-2015), and mice were maintained in the animal facility within the Department of Nuclear Medicine at the University Hospital rechts der Isar according to institutional guidelines. For the tumor implantation, mice were anesthetized under isoflurane flow 3–5% (v/v). Each animal was injected subcutaneously with 5 × 10^6^ LNCaP cells in 100 µl serum-free RPMI medium mixed with 100 µl matrigel (BD Biosciences, Germany) in the right shoulder region. Animals with tumors above a diameter of 0.5 cm were used for the experiments.

### Radiolabeling procedures and quality controls

rhPSMA-10.1 and rhPSMA-10.2 were radiolabeled with non-carrier-added lutetium-177 according to established procedures (Benesova et al. 2015; Weineisen et al. 2015). Briefly, 12.5 nmol of the precursor was dissolved in DMSO to a concentration of 1 mg/mL and filled up to 400 μL using 0.4 M acetate buffer and gentisic acid (10 mg/mL) at pH 5.5. 500 MBq of [^177^Lu]LuCl_3_ in 0.04 M HCl (ITM Medical Isotope GmbH, Garching, Munich, Germany) were added to this solution and incubated at 90 °C for 30 min. Radionuclide incorporation (RNI) was determined via radio-thin layer chromatography (TLC), using glass microfiber paper impregnated with silicic acid (Agilent Technologies, Santa Clara, US) and 0.1 M sodium citrate at pH 5.0 as mobile phase. Samples were read-out on the MiniScan scanner from Bioscan (Eckert and Ziegler, Brussels, Belgium). Radiochemical purity (RCP) was further evaluated via radio high-performance liquid chromatography (HPLC) on a Prominence system with a Photo Diode Array detector (Shimadzu, Kyoto, Japan) and a GABI Star detector (Raytest, Straubenhardt, Germany). Eluents for all HPLC operations were water (solvent A) and acetonitrile (solvent B), both containing 0.1% trifluoroacetic acid. An XTerra MS C18 OBD column (Waters, Germany) was used with a linear gradient of 15–95% B in 20 min, followed by 95% B for 10 min.

### Stability in vitro

For the assessment of the isomers in vitro stability, freshly produced [^177^Lu]Lu-rhPSMA-10.1 and [^177^Lu]Lu-rhPSMA-10.2 were aliquoted in four micro reaction vials and diluted 1:10 in acetate buffer (0.4 M, pH 5.5, 10 mg/mL gentisic acid), PBS pH 7.4, 0.9% NaCl solution, and human serum, then stored at 37 °C up to 168 h. At different time points (1, 24, 48, 72, and 168 h), samples were analyzed via TLC.

### Biodistribution studies

For each radiotherapeutic agent two cohorts of animals were used, one with non-tumor-bearing SCID mice (n = 2, each time point) and one with LNCaP tumor-bearing SCID mice (n = 4–5, each time point). Mice were anesthetized by isoflurane inhalation and maintained under continuous isoflurane anesthesia (1.75–3% v/v), while 1.6 MBq (~ 40 pmol) of radioligand were injected in the tail vein using a catheter in a total volume of 200 µl saline solution. Non-tumor bearing animals were sacrificed at 1, 12, 24, 48, and 168 h post injection (p.i.), while the tumor-bearing animals were sacrificed at 1, 24, and 168 h p.i. Tumor, blood, and other selected tissues were excised, collected, weighted, and then measured in a gamma-counter (2480 WIZARD2, PerkinElmer, Waltham, US). Radioactivity uptake was calculated as a percentage of the injected dose per gram of tissue (%ID/g) using a 1% (v/v) standard of the injected activity.

### Dosimetry calculations

Five time points were employed to calculate the Time-Integrated Activity Coefficients (TIAC) for each radiopharmaceutical. The activity accumulation in significant source organs was determined using both numerical integration and physical decay, as per Yuan et al. (1993). We estimated radiation doses to normal organs in a standard 70 kg adult model by using time-dependent organ activity concentrations (expressed as %ID/g) and total-body activities.

Tissue activity concentrations in mice were converted to fractional activities in the standard 70 kg adult by considering the relative organ masses between the standard adult and the 25 g mouse. The time-dependent total-body activity was modelled using an exponential function and assumed that the difference between the injected activity and total-body activity was excreted through urine, as liver and gastrointestinal tract activity concentrations remained consistently low throughout. Residence times were computed as the difference between the total-body residence time and the sum of the organ and urine residence times. Finally, the absorbed doses (in mGy/MBq) in organs of a standard adult were calculated using OLINDA/EXM1.0 (Stabin et al. 2005). For estimating tumor doses the unit density sphere model described by Stabin and Konijnenberg (2000) was utilized.

### Statistics

All data are presented as mean ± standard deviation (SD). Statistical analyses were performed with GraphPad Prism 4.0 software using Student’s t-test for unpaired data. Two-sided significance levels were calculated and *P* < 0.05 values were considered statistically significant.

## Results

### Radiochemistry and stability

After radiolabeling of the rhPSMA-10 isomers according to the previously published procedures for DOTA-conjugated ligands (Benesova et al. 2015; Weineisen et al. 2015), analysis via radio-TLC revealed a high RNI of > 99.5%. RCP as determined by radio-HPLC was 94.2 ± 0.2% for [^177^Lu]Lu-rhPSMA-10.1 and 94.5 ± 0.2 [^177^Lu]Lu-rhPSMA-10.2 with retention times of 14.2 min and 13.8 min, respectively. A hydrophilic impurity eluting approximately 2.3 min before the product was identified as the main side product (relative area under the curve: 5%). Representative HPLC and TLC chromatograms have been included into the supporting information (Additional file 1: Figures S1 and S2). Both ligands were produced in a molar activity of 38.0 GBq/µmol.

**Fig. 2:**
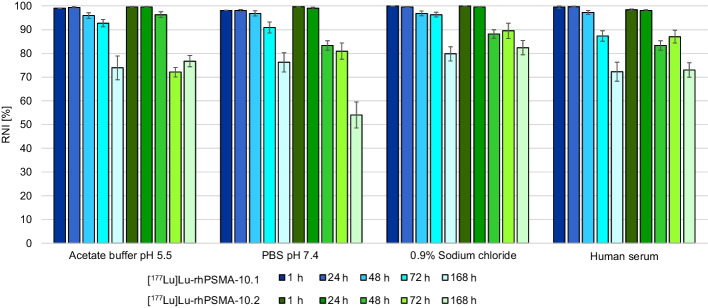
Stability of [^177^Lu]Lu-rhPSMA-10.1 (blue) and [^177^Lu]Lu-rhPSMA-10.2 (green) in acetate buffer (pH 5.5), PBS pH 7.4, 0.9% sodium chloride and human serum at 37 °C. Radionuclide incorporation (RNI; %) was determined in triplicate via radio-TLC after incubation for 1 to 168 h

Both [^177^Lu]Lu-rhPSMA-10.1 and [^177^Lu]Lu-rhPSMA-10.2 isomers showed high and comparable stability > 98% for 24 h in all media (Fig. 2). After longer incubation times up to 168 h, the RNI decreased for both isomers to approximately 72–80% for [^177^Lu]Lu-rhPSMA-10.1 and 54–82% for [^177^Lu]Lu-rhPSMA-10.2. The highest stability for both isomers was found in 0.9% sodium chloride (80 ± 3% for 10.1 and 82 ± 3% for 10.2 after 168 h). In human serum both isomers showed a stability of approximately 70% after 168 h (see Additional file 1: Table S1 for absolute values).

### Biodistribution and dosimetry in non-tumor-bearing SCID mice

**Fig. 3:**
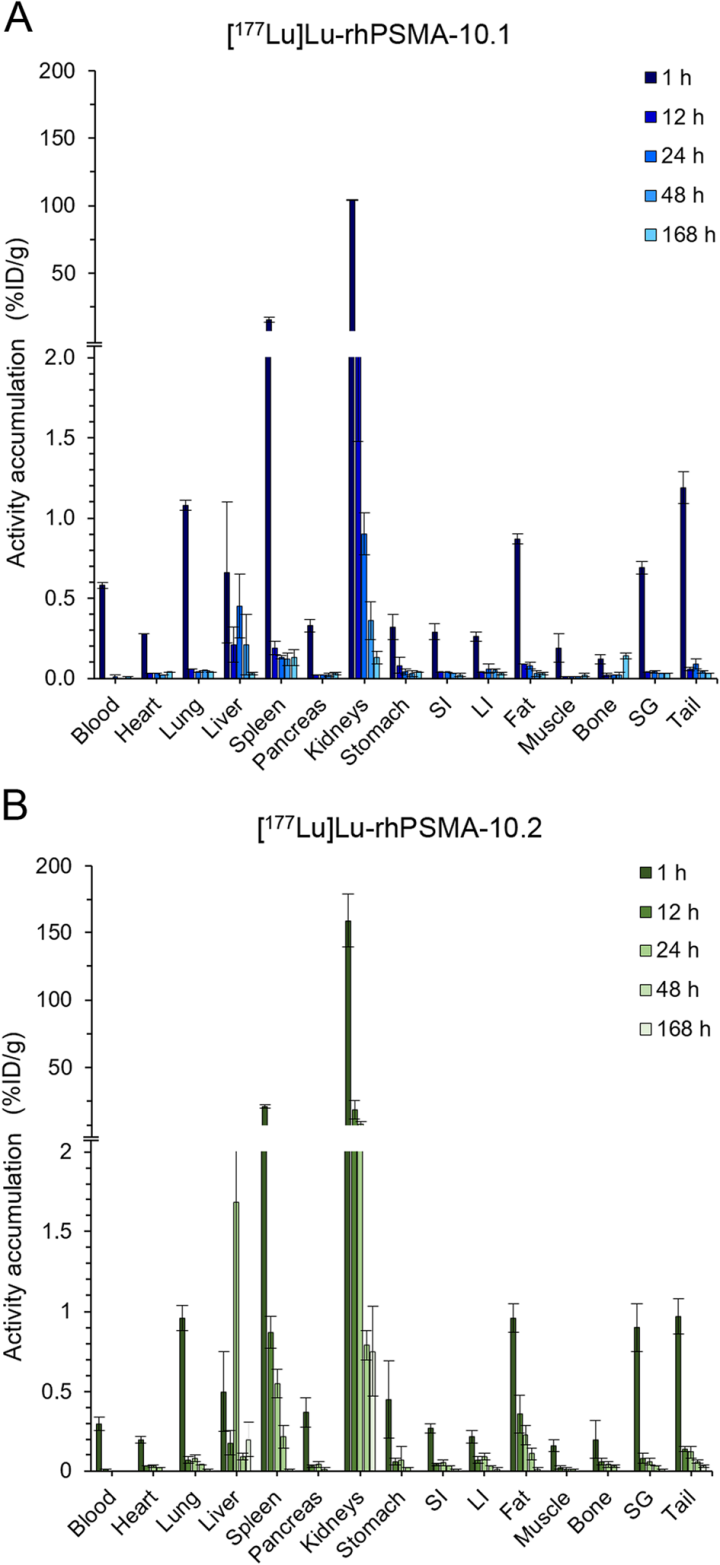
Biodistribution studies of [^177^Lu]Lu-rhPSMA-10.1 (**A**) and [^177^Lu]Lu-rhPSMA-10.2 (**B**) in non-tumor-bearing mice at 1, 12, 24 and 168 h p.i. Values are expresses as a percentage of injected dose per gram (%ID/g), mean ± SD (n = 2). SI, small intestine; LI, large intestine; SG, salivary glands

In non-tumor-bearing mice, [^177^Lu]Lu-rhPSMA-10.1 and [^177^Lu]Lu-rhPSMA-10.2 displayed fast pharmacokinetics with excretion occurring primarily via the renal system (Fig. 3, Additional file 1: Table S2). Most of the radioactivity was cleared at 1 h p.i. in most of the analyzed tissues, organs and body fluids ≤ 1.4%ID/g. Higher tracer accumulation was observed in the kidneys and spleen, which then reduced over the following 24 to 48 h. Remarkable differences in the accumulation of both isomers in the kidneys were noted, with the *D*-Dap isomer [^177^Lu]Lu-rhPSMA-10.1 displaying a 1.5-fold lower initial kidney uptake of 104.2 ± 0.2%ID/g at 1 h, compared to the *L*-Dap isomer [^177^Lu]Lu-rhPSMA-10.2 (159.4 ± 20.0%ID/g). The kidney clearance was found to be faster for [^177^Lu]Lu-rhPSMA-10.1 (0.9 ± 0.1%ID/g at 24 h and 0.4 ± 0.1%ID/g at 48 h), compared to [^177^Lu]Lu-rhPSMA-10.2 (8.2 ± 1.6%ID/g at 24 h and 0.8 ± 0.1%ID/g at 48 h). Uptake in murine salivary glands was comparable at 1 h p.i. for both agents (0.7 ± 0.04%ID/g vs. 0.9 ± 0.2%ID/g for 10.1 vs. 10.2), with fast excretion during the first 12 h (< 0.1%ID/g). The total body effective doses were 0.018 mSv/MBq (1 h voiding interval; Additional file 1: Table S3) and 0.023 mSv/MBq (3.5 h; Additional file 1: Table S4) for [^177^Lu]Lu-rhPSMA-10.1 and 0.052 mSv/MBq (1 h, Additional file 1: Table S5) and 0.056 mSv/MBq (3.5 h, Additional file 1: Table S6) for [^177^Lu]Lu-rhPSMA-10.2, respectively. Kidneys and spleen were the tissues with a significant accumulation and therefor used as source organs for the model.

### Biodistribution and dosimetry in tumor-bearing mice

**Fig. 4:**
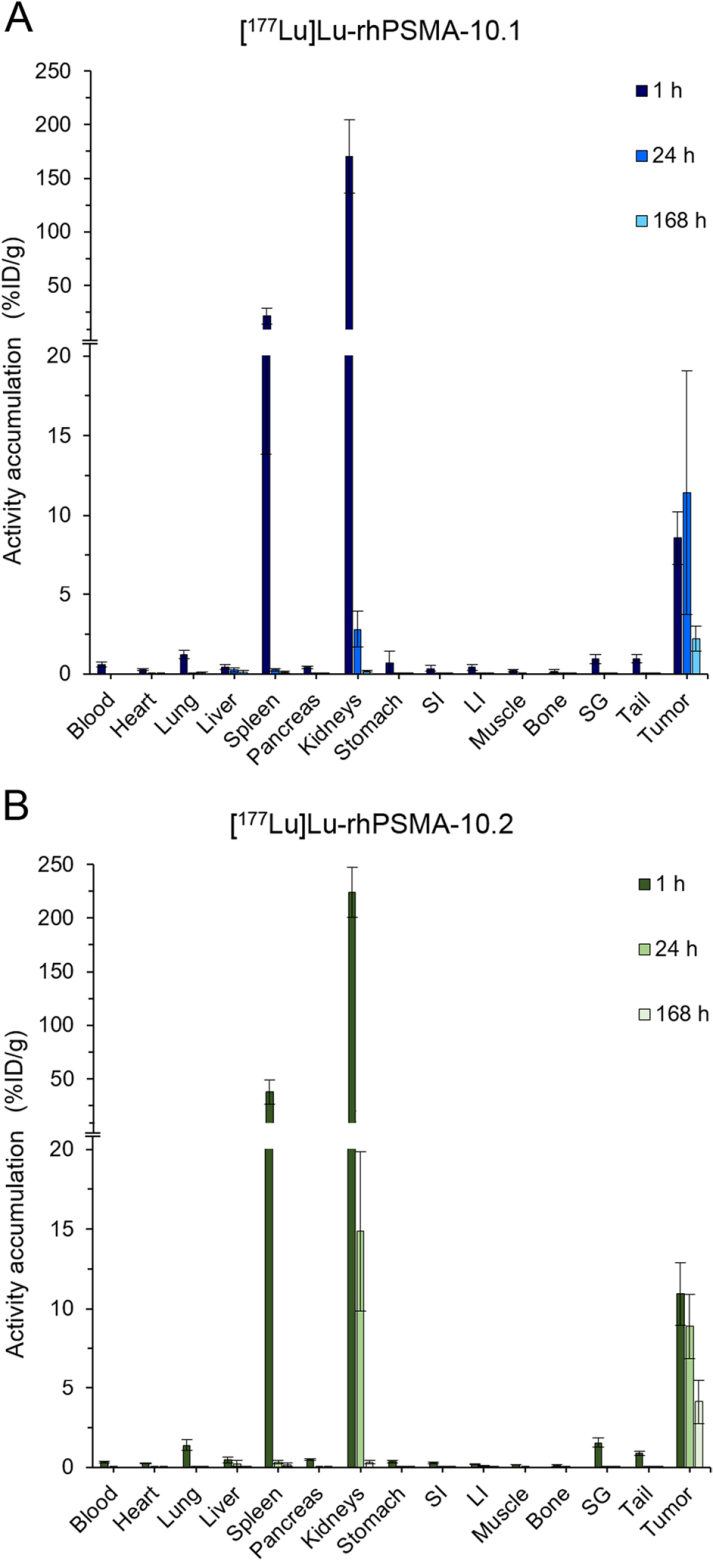
Biodistribution studies of [^177^Lu]Lu-rhPSMA-10.1 (**A**) and [^177^Lu]Lu-rhPSMA-10.2 (**B**) in mice bearing LNCaP tumors at 1, 24 and 168 h p.i. Values are expresses as a percentage of injected dose per gram (%ID/g), mean ± SD (n = 4–5). SI, small intestine; LI, large intestine; SG, salivary glands

In LNCaP tumor-bearing mice [^177^Lu]Lu-rhPSMA-10.1 and [^177^Lu]Lu-rhPSMA-10.2 displayed an accumulation pattern in normal organs similar to that observed in non-tumor bearing mice, with fast clearance via the kidneys. A high tumor uptake equal to 8.6 ± 1.7%ID/g and 10.9 ± 2.0%ID/g at 1 h p.i. was measured for isomer [^177^Lu]Lu-rhPSMA-10.1 and [^177^Lu]Lu-rhPSMA-10.2, respectively (Fig. 4, Additional file 1: Table S7). Over 24 h the tumor uptake remained constant, decreasing over 168 h to a level of 2.2 ± 0.8 and 4.1 ± 1.4%ID/g for isomer 10.1 and 10.2, respectively. As observed in non-tumor bearing mice, [^177^Lu]Lu-rhPSMA-10.1 was cleared more rapidly from the kidneys, expressed by an approximately 1.3-fold and fivefold lower accumulation compared with [^177^Lu]Lu-rhPSMA-10.2 at 1 h and 24 h p.i., respectively. The total body effective doses were 0.012 mSv/MBq (1 h voiding interval) and 0.029 mSv/MBq (3.5 h) for [^177^Lu]Lu-rhPSMA-10.1 and 0.018 mSv/MBq (1 h) and 0.026 mSv/MBq (3.5 h) for [^177^Lu]Lu-rhPSMA-10.2, respectively. Tumor doses were quite comparable with an about 12% higher dose for [^177^Lu]Lu-rhPSMA-10.2 (Additional file 1: Figure S3).

## Discussion

The PSMA binding pocket is reported to tolerate a variety of large structural modifications, e.g., fluorescent dyes (Derks et al. 2019), albumin binding groups (Kuo et al. 2021; Deberle et al. 2020) or silicon–fluoride acceptors (Wurzer et al. 2020a). However, we previously demonstrated that the stereoconfiguration of the linker has a strong influence on the PSMA binding characteristics and in vivo behavior (Wurzer et al. 2020b). In a preclinical selection process of rhPSMA-7 isomers which are different in the stereoconfiguration of a Diaminopropionic acid unit (*D*-Dap or *L*-Dap) and the chelator (*R*-DOTAGA or *S*-DOTAGA), rhPSMA-7.3 (*D*-Dap, S-DOTAGA) displayed superior tumor uptake and faster clearance from background organs, compared with other isomers (Wurzer et al. 2020b). Importantly, these findings were confirmed in a retrospective clinical analysis in which [^18^F]Ga-rhPSMA-7.3 displayed lower excretion into the bladder (SUV_mean_ 2.0 vs. 4.0), lower kidney uptake (32.4 vs. 35.7), and higher tumor uptake (32.5 ± 42.7 vs. 20.0 ± 20.2) (Knorr et al. 2022), compared to diastereomeric mixture, [^18^F]Ga-rhPSMA-7.

The subsequent development of a ^177^Lu-labeled rhPSMA ligand for radioligand therapy aimed to accelerate the clearance from the kidneys and maintain the tumor uptake (Wurzer et al. 2022). These efforts resulted in the development of rhPSMA-10.1 and -10.2, two DOTA-based analogues of rhPSMA-7 with a lower overall negative charge and a lower binding strength to HSA compared with the former lead. In order to carefully compare the in vivo behavior of [^177^Lu]Lu-rhPSMA-10.1 and -10.2 we performed the present biodistribution study in healthy and tumor-bearing mice over a 168 h time course. The injected mass of each isomer (0.04 nmol, 0.06 µg for a 25 g mouse) corresponds to a typical dose of [^177^Lu]Lu-PSMA-617 (150–250 µg, 144–240 nmol, 75 kg patient), administered during the VISION trial (Sartor et al. 2021).

After radiolabeling of rhPSMA-10.1 and -10.2 with lutetium-177, formation of a hydrophilic impurity (relative area: 5%) was observed by radio-HPLC. In the context of clinical development, the hydrophilic impurity was identified as the hydrolyzed silicon–fluoride acceptor (SiOH), whereupon an optimized radiolabeling procedure was developed, resulting in an improved RCP ≥ 97%. In different formulations a comparable and high stability was found for both isomers over 24 h, allowing their centralized production and distribution. The reason for the different stability at later time points warrants further investigation in future studies. In the comparative biodistribution studies, the two ^177^Lu-labeled isomers displayed the typical distribution pattern of PSMA-targeted radioligands, with fast clearance kinetics from the blood pool and background tissues and high tumor uptake. The high uptake in murine spleen at 1 h p.i., has also been observed previously for a variety of other PSMA-targeted ligands and was found to be blockable by excess of a competitor (Benesova et al. 2015; Schottelius et al. 2019).

In PSMA-targeted radioligand therapy, the kidney and the bone marrow are still considered the main organs at risk, and the uptake in these tissues should be carefully considered (Yordanova et al. 2017). Because of the increasing interest in using [^177^Lu]Lu-PSMA ligands in early disease stages (e.g. PSMAfore and PSMAddition trials), especially the tumor-to-kidney ratio is one of the most important selection criteria for novel radioligands in order to reduce potential renal side effects in men with several years of life expectancy.

**Fig. 5:**
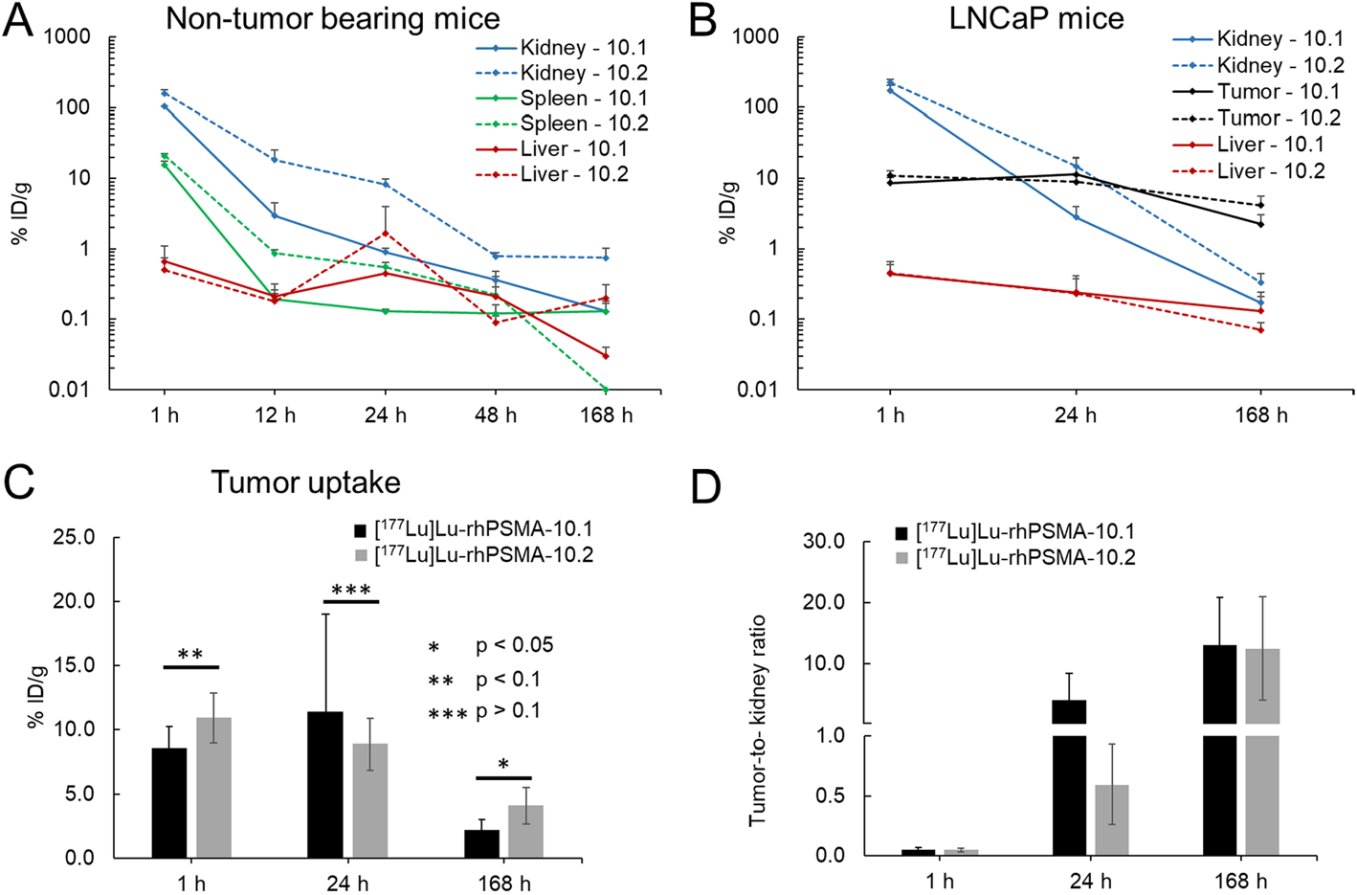
Time activity curves of [^177^Lu]Lu-rhPSMA-10.1 (solid line) and -10.2 (dashed line), expressed as a percentage of the injected dose per gram (%ID/g) between 1 and 168 h p.i. in **A** in kidneys (blue), spleen (green) and liver (red) of non-tumor bearing mice and **B** in kidneys (blue), liver (red) and tumor (black) of mice bearing LNCaP tumors. **C** Tumor uptake (%ID/g) of [^177^Lu]Lu-rhPSMA-10.1 (black) and -10.2 (grey) at 1, 24 and 168 h and determination of respective tumor-to-kidney ratios (**D**)

Despite the structural similarity of both ligands, [^177^Lu]Lu-rhPSMA-10.1 was found to display significantly lower initial accumulation in the kidneys at 1 h p.i. and faster excretion kinetics, compared with the *L*-Dap-based [^177^Lu]Lu-rhPSMA-10.2. This is demonstrated by the ninefold and fivefold lower kidney uptake at 24 h p.i. in non-tumor- and tumor-bearing mice, respectively (Fig. 5A, B). Our findings support the results from the previously performed in vivo single-time point comparison of [^177^Lu]Lu-rhPSMA-10.1 and -10.2, which was conducted at 24 h p.i. (Wurzer et al. 2022). Here, [^177^Lu]Lu-rhPSMA-10.1 showed a fourfold lower accumulation in the kidneys, compared to [^177^Lu]Lu-rhPSMA-10.2, while tumor uptake was comparable (9.8 ± 0.3 vs. 10.5 ± 3.3%ID/g for 10.1 vs. 10.2) (Wurzer et al. 2022).

While no statistically significant difference in the tumor uptake was found at early time points for both radiopharmaceuticals, [^177^Lu]Lu-rhPSMA-10.2 showed improved tumor retention, expressed by an approximately twofold higher uptake after 168 h p.i. Due to the fast kidney clearance of [^177^Lu]Lu-rhPSMA-10.1, the tumor-to-kidney values were found to be superior for [^177^Lu]Lu-rhPSMA-10.1 after 24 h and similar for both ligands after 168 h. In the previous in vitro characterization of [^177^Lu]Lu-rhPSMA-10.1 versus -10.2, both displayed a similar PSMA binding affinity (2.8 nM vs. 3.6 nM) and internalization rate (177% vs. 206%; expressed as a percentage of the reference ligand [^125^I]IBA-KuE) (Wurzer et al. 2022). Moreover, the isomers were found to have an identical lipophilicity (logP: − 3.8 for both; distribution coefficient in octanol and PBS pH 7.4), and binding strength to HSA was in a similar range (Wurzer et al. 2022). Based on these characteristics, it remains challenging to explain the observed differences in the kidney accumulation of both ligands and the higher tumor retention of [^177^Lu]Lu-rhPSMA-10.2 at 168 h p.i.. Given that both isomers exhibited comparable complex stability in human serum over a period of 168 h, the observed differences in biodistribution are likely attributed to distinct pharmacokinetics rather than different amounts of unbound lutetium-177.

These findings elucidate once again that there are potentially other parameters, influencing the biodistribution of radioligands which are currently not assessed in routine screening experiments, like for example differences in magnitude and binding affinities to plasma proteins other than HSA, such as α-1-acid glycoprotein (Smith and Waters 2018), transthyretin (Buxbaum and Reixach 2009), or lipoproteins (Wasan et al. 2008). Moreover, species differences between mouse serum albumin and HSA need to be considered (Roopenian et al. 2015).

In a recently performed preclinical biodistribution study of [^177^Lu]Lu-PSMA I&T in the same xenograft models, the reference shows a similar clearance from background tissues but slower renal clearance kinetics, compared to the radiohybrid ligands in the present study (Yusufi et al. 2021). While initial kidney uptake is in a similar range at 1 h p.i. (166–224%ID/g), the accelerated clearance kinetics of radiohybrid ligands result in a 26-fold and fivefold lower kidney uptake at 24 h p.i. for [^177^Lu]Lu-rhPSMA-10.1 and -10.2, respectively, compared to the reference. The tumor uptake of both isomers is superior at all time points, compared to [^177^Lu]Lu-PSMA I&T: 4.4 ± 1.5%ID/g at 1 h, 6.2 ± 0.1%ID/g at 24 h and 1.0 ± 0.2%ID/g at 168 h.Despite the promising pharmacokinetics of [^177^Lu]Lu-rhPSMA-10.1, it is in general questionable to what extent these data, especially murine kidney uptake, is transferable to humans. Data reported for the recently FDA-approved [^177^Lu]Lu-PSMA-617 perfectly illustrate the pitfalls of murine kidney uptake associated with PSMA-ligands. While [^177^Lu]Lu-PSMA-617 showed a 25-fold lower preclinical kidney uptake compared with [^177^Lu]Lu-PSMA I&T (1.4 ± 0.4 vs. 34.7 ± 17.2%ID/g at 24 h p.i.) (Wurzer et al. 2022), Baum et al. impressively demonstrated similar renal half-lives, resulting in nearly identical absorbed radiation doses (0.8 vs. 0.9 Gy/GBq for 617 vs. I&T) in patients (Schuchardt et al. 2022). Further evaluations will be required to fully understand species differences of PSMA-targeted ligands. Until the availability of improved preclinical methods to predict human pharmacokinetics more accurately, especially in organs at risk, murine uptake values are still the most important selection criteria for clinical translation of novel radiopharmaceuticals. Data from ongoing clinical investigations will be valuable to retrospectively judge the results obtained in preclinical studies in mice.

## Conclusion

Our preclinical data on biodistribution and dosimetry indicate a more favorable profile of [^177^Lu]Lu-rhPSMA-10.1 compared to [^177^Lu]Lu-rhPSMA-10.2 for PSMA targeting RLT. [^177^Lu]Lu-rhPSMA-10.1 shows fast kidney clearance kinetics resulting in excellent tumor-to-organ ratios over a therapy relevant time course. [^177^Lu]Lu-rhPSMA-10.1 is currently being investigated in clinical phase I/II studies in patients with mCRPC (NCT05413850), in patients with high-risk localized PC (NCT06066437, Nautilus Trial) and after external beam radiotherapy (NCT06105918).

### Supplementary Information

**Additional file 1.** Supporting Information is provided in addition to data presented in the main manuscript, including representative chromatograms and absolute values of biodistribution and dosimetry data.

## Data Availability

The datasets used and analyzed during the current study are available from the corresponding author on reasonable request.

## Abbreviations

%ID/g: Percentage of injected dose per gram
DAP: 2,3-Diaminopropionic acid
DMSO: Dimethyl sulfoxide
DOTA: 2,2′,2′′,2′′′-(1,4,7,10-Tetraazacyclododecane-1,4,7,10-tetrayl)tetraacetic acid
DOTAGA: 2-(4,7,10-Tris(carboxymethyl)-1,4,7,10-tetraazacyclododecan-1-yl)pentanedioic acid
HPLC: High-performance liquid chromatography
HSA: Human serum albumin
mCRPC: Metastatic castration-resistant prostate cancer
PBS: Phosphate-buffered saline
PET: Positron emission tomography
p.i.: Post injection
PSMA: Prostate-specific membrane antigen
rh: Radiohybrid
RLT: Radioligand therapy
RNI: Radionuclide incorporation
SCID: Severe combined immunodeficiency disease
SD: Standard deviation
SiFA: Silicon–fluoride acceptor
TIAC: Time-integrated activity coefficients
TLC: Thin-layer chromatography
