# Supplementary material for: Preclinical comparison of [177Lu]Lu-rhPSMA-10.1 and [177Lu]Lu-rhPSMA-10.2 for endoradiotherapy of prostate cancer: biodistribution and dosimetry studies
Source: EJNMMI Radiopharm Chem. 2024 Feb 26;9:18. doi: 10.1186/s41181-024-00246-2 (PMC10897098; doi:10.1186/s41181-024-00246-2)
Supplement: Supplementary file 1 — Additional file 1. Supporting Information is provided in addition to data presented in the main manuscript, including representative chromatograms and absolute values of biodistribution and dosimetry data. [file 41181_2024_246_MOESM1_ESM.docx]

**Preclinical Comparison of [^177^Lu]Lu-rhPSMA-10.1 and [^177^Lu]Lu-rhPSMA-10.2 for Endoradiotherapy of Prostate Cancer: Biodistribution and Dosimetry Studies**

**Supporting Information**

**
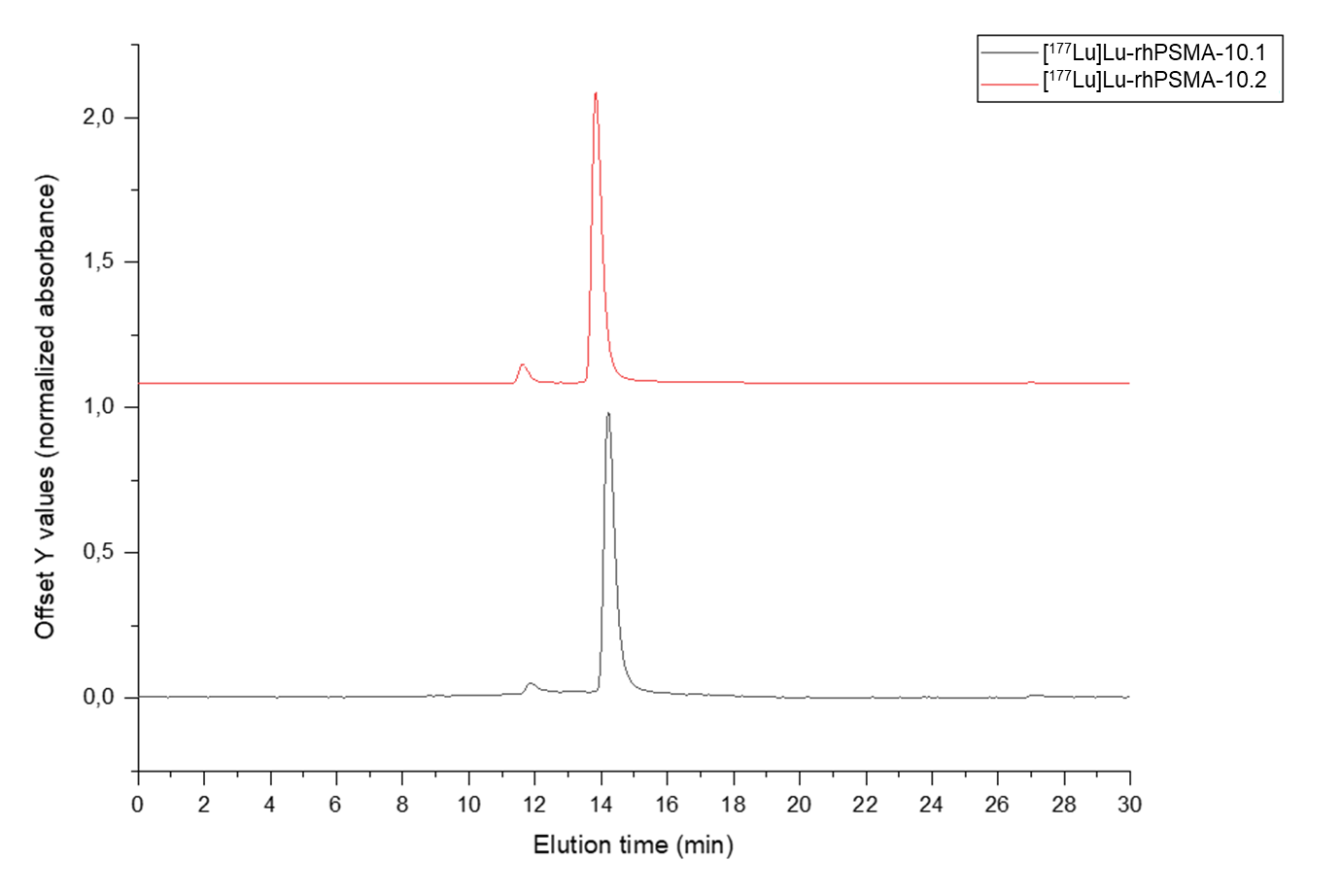
**

**Supplemental Figure 1.** Representative HPLC radio-chromatogram of [^177^Lu]Lu-rhPSMA-10.1 (black) and [^177^Lu]Lu-rhPSMA-10.2 (red) for quality control, recorded on a Prominence system (Shimadzu, Kyoto, Japan) with a GABI Star detector (Raytest, Straubenhardt, Germany). Eluents for all HPLC operations were water (solvent A) and acetonitrile (solvent B), both containing 0.1% trifluoroacetic acid. An XTerra MS C18 OBD column (Waters, Germany) was used with a linear gradient of 15-95%B in 20 minutes, followed by 95%B for 10 minutes.


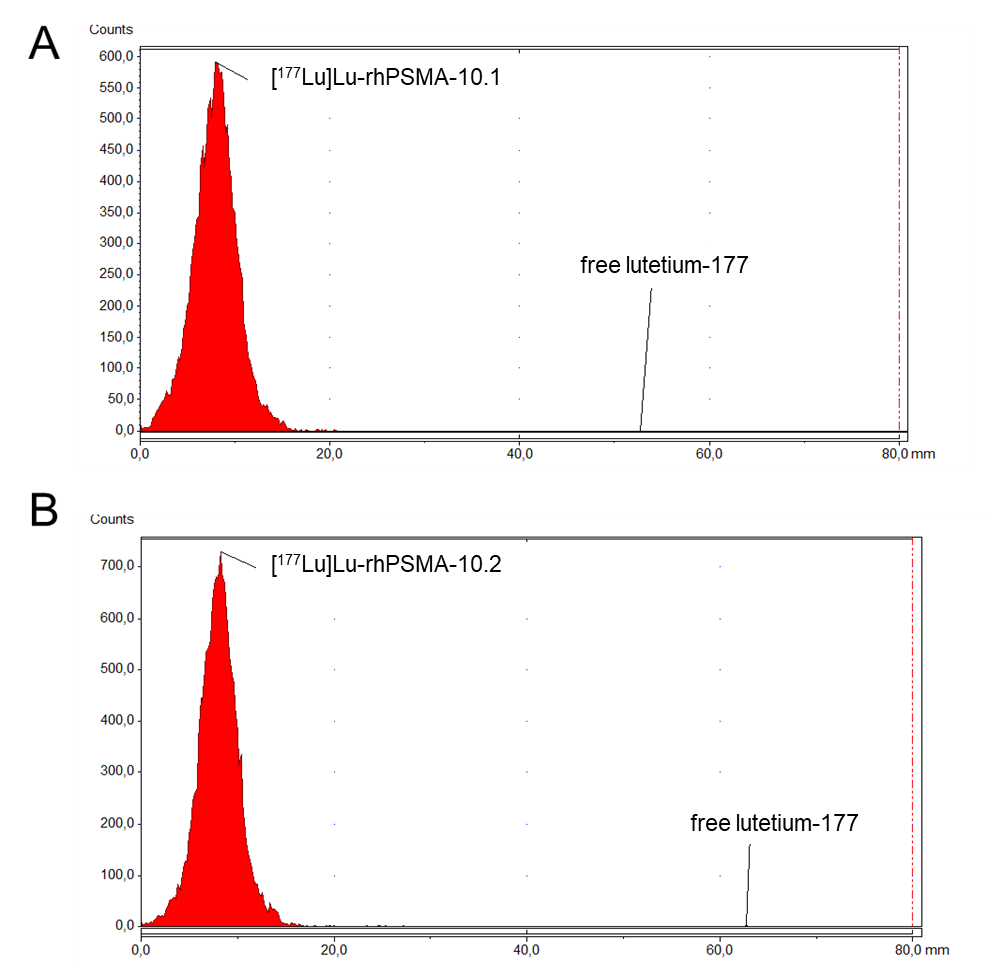


**Supplemental Figure 2.** Representative radio-TLC scan of [^177^Lu]Lu-rhPSMA-10.1 (A) and [^177^Lu]Lu-rhPSMA-10.2 (B) using glass microfiber paper impregnated with silicic acid (Agilent Technologies, Santa Clara, US) and 0.1 M sodium citrate at pH 5.0 as mobile phase.

**Supplemental Table 1.** Stability of ^177^Lu-rhPSMA-10.1 and ^177^Lu-rhPSMA-10.2 in acetate buffer (pH 5.5), PBS pH 7.4, 0.9% sodium chloride and human serum at 37°C. Radionuclide Incorporation (RNI; %) was determined in triplicate via radio-TLC after incubation for 1 to 168 h.

**Individual Measurements:**

| **[^177^Lu]Lu-rhPSMA-10.1** | RNI (%) at different time points | | | | |
| --- | --- | --- | --- | --- | --- |
|  | 1 h | 24 h | 48 h | 72 h | 168 h |
| Acetate buffer pH 5.5 | 98.89 | 99.64 | 94.60 | 94.36 | 70.15 |
|  | 99.09 | 99.19 | 96.79 | 92.29 | 71.99 |
|  | 99.05 | 99.17 | 96.36 | 91.40 | 79.66 |
| PBS pH 7.4 | 98.15 | 98.00 | 95.84 | 88.47 | 72.02 |
|  | 98.05 | 98.49 | 98.02 | 91.10 | 80.10 |
|  | 97.99 | 97.40 | 96.52 | 93.04 | 76.58 |
| 0.9% Sodium Chloride | 99.87 | 99.31 | 97.00 | 95.15 | 79.17 |
|  | 99.70 | 99.71 | 95.70 | 96.95 | 77.16 |
|  | 99.99 | 99.49 | 97.66 | 96.70 | 83.00 |
| Human serum | 99.10 | 99.65 | 98.01 | 88.99 | 67.73 |
|  | 99.99 | 99.77 | 97.19 | 84.82 | 75.13 |
|  | 99.26 | 99.30 | 96.44 | 88.00 | 74.00 |

| **[^177^Lu]Lu-rhPSMA-10.2** | RNI (%) at different time points | | | | |
| --- | --- | --- | --- | --- | --- |
|  | 1 h | 24 h | 48 h | 72 h | 168 h |
| Acetate buffer pH 5.5 | 99.30 | 99.37 | 95.15 | 69.89 | 77.70 |
|  | 99.62 | 99.73 | 97.53 | 72.35 | 78.58 |
|  | 99.61 | 99.35 | 96.27 | 73.96 | 73.98 |
| PBS pH 7.4 | 99.65 | 99.45 | 81.04 | 76.99 | 48.25 |
|  | 99.71 | 98.85 | 84.79 | 83.12 | 59.10 |
|  | 99.49 | 98.76 | 84.17 | 82.69 | 54.52 |
| 0.9% Sodium Chloride | 99.89 | 99.66 | 88.62 | 91.95 | 81.77 |
|  | 99.72 | 99.52 | 86.23 | 85.89 | 79.80 |
|  | 99.99 | 99.38 | 89.65 | 90.56 | 85.67 |
| Human serum | 97.87 | 98.01 | 85.51 | 84.97 | 69.57 |
|  | 98.63 | 98.30 | 82.75 | 85.97 | 75.13 |
|  | 98.31 | 97.60 | 81.55 | 90.14 | 74.38 |

**Mean Values:**

| [^177^Lu]Lu-rhPSMA-10.1 | RNI (%) | | | | |
| --- | --- | --- | --- | --- | --- |
|  | 1 h | 24 h | 48 h | 72 h | 168 h |
| Acetate buffer pH 5.5 | 99.0±0.1 | 99.3±0.3 | 95.9±1.2 | 92.7±1.5 | 73.9±5.0 |
| PBS pH 7.4 | 98.1±0.1 | 98±0.5 | 96.8±1.1 | 90.9±2.3 | 76.2±4.0 |
| 0.9% Sodium Chloride | 99.9±0.2 | 99.5±0.2 | 96.8±1.0 | 96.3±1.0 | 79.8±3.0 |
| Human serum | 99.5±0.5 | 99.6±0.2 | 97.2±0.8 | 87.3±2.2 | 72.3±4.0 |

| [^177^Lu]Lu-rhPSMA-10.2 | RNI (%) | | | | |
| --- | --- | --- | --- | --- | --- |
|  | 1 h | 24 h | 48 h | 72 h | 168 h |
| Acetate buffer pH 5.5 | 99.5±0.2 | 99.5±0.2 | 96.3±1.2 | 72.1±2.0 | 76.7±2.4 |
| PBS pH 7.4 | 99.6±0.1 | 99±0.4 | 83.3±2.0 | 80.9±3.4 | 54±5.5 |
| 0.9% Sodium Chloride | 99.9±0.1 | 99.5±0.1 | 88.2±1.8 | 89.5±3.2 | 82.4±3.0 |
| Human serum | 98.3±0.4 | 98±0.3 | 83.3±2.0 | 87±2.7 | 73±3.0 |

**Supplemental Table 2.** Biodistribution studies of [^177^Lu]Lu-rhPSMA-10.1 and [^177^Lu]Lu-rhPSMA-10.2 in healthy mice at 1, 12, 24, 48 and 168 h p.i. Values are expressed as a percentage of injected dose per gram (%ID/g), mean ± SD (n = 2). SI: small intestine; LI: large intestines; SG: salivary glands.

| Radioligand | [^177^Lu]Lu-rhPSMA-10.1 | | | | | | | | | | [^177^Lu]Lu-rhPSMA-10.2 | | | | | | | | | |
| --- | --- | --- | --- | --- | --- | --- | --- | --- | --- | --- | --- | --- | --- | --- | --- | --- | --- | --- | --- | --- |
| Time point | 1 h | | 12 h | | 24 h | | 48 h | | 168 h | | 1 h | | 12 h | | 24 h | | 48 h | | 168 h | |
|  | Mean %ID/g | ±SD | Mean %ID/g | ±SD | Mean %ID/g | ±SD | Mean %ID/g | ±SD | Mean %ID/g | ±SD | Mean %ID/g | ±SD | Mean %ID/g | ±SD | Mean %ID/g | ±SD | Mean %ID/g | ±SD | Mean %ID/g | ±SD |
| Blood | 0.58 | 0.02 | 0.00 | 0.00 | 0.01 | 0.01 | 0.00 | 0.00 | 0.01 | 0.00 | 0.30 | 0.04 | 0.01 | 0.00 | 0.00 | 0.00 | 0.00 | 0.00 | 0.00 | 0.00 |
| Heart | 0.28 | 0.00 | 0.03 | 0.00 | 0.03 | 0.00 | 0.02 | 0.00 | 0.04 | 0.00 | 0.20 | 0.02 | 0.03 | 0.00 | 0.03 | 0.01 | 0.02 | 0.00 | 0.00 | 0.00 |
| Lung | 1.08 | 0.03 | 0.06 | 0.00 | 0.04 | 0.00 | 0.05 | 0.00 | 0.04 | 0.00 | 0.96 | 0.08 | 0.07 | 0.02 | 0.08 | 0.02 | 0.04 | 0.00 | 0.01 | 0.00 |
| Liver | 0.66 | 0.44 | 0.21 | 0.11 | 0.45 | 0.20 | 0.21 | 0.19 | 0.03 | 0.01 | 0.50 | 0.25 | 0.18 | 0.08 | 1.68 | 1.20 | 0.09 | 0.02 | 0.20 | 0.11 |
| Spleen | 15.63 | 2.05 | 0.19 | 0.04 | 0.13 | 0.01 | 0.12 | 0.04 | 0.13 | 0.05 | 21.12 | 1.20 | 0.87 | 0.10 | 0.55 | 0.09 | 0.22 | 0.07 | 0.01 | 0.00 |
| Pancreas | 0.33 | 0.04 | 0.02 | 0.00 | 0.02 | 0.00 | 0.02 | 0.01 | 0.03 | 0.01 | 0.37 | 0.09 | 0.03 | 0.01 | 0.04 | 0.02 | 0.01 | 0.01 | 0.00 | 0.00 |
| Kidneys | 104.17 | 0.27 | 2.97 | 1.49 | 0.90 | 0.13 | 0.36 | 0.12 | 0.13 | 0.04 | 159.37 | 20.04 | 18.40 | 6.89 | 8.21 | 1.62 | 0.79 | 0.09 | 0.75 | 0.28 |
| Stomach | 0.32 | 0.08 | 0.08 | 0.05 | 0.04 | 0.02 | 0.03 | 0.02 | 0.04 | 0.00 | 0.45 | 0.24 | 0.06 | 0.02 | 0.07 | 0.09 | 0.02 | 0.00 | 0.00 | 0.00 |
| SI | 0.29 | 0.05 | 0.04 | 0.00 | 0.04 | 0.00 | 0.03 | 0.00 | 0.02 | 0.01 | 0.27 | 0.03 | 0.04 | 0.01 | 0.05 | 0.02 | 0.03 | 0.00 | 0.01 | 0.00 |
| LI | 0.26 | 0.03 | 0.04 | 0.00 | 0.06 | 0.03 | 0.05 | 0.01 | 0.03 | 0.01 | 0.22 | 0.04 | 0.07 | 0.02 | 0.09 | 0.02 | 0.03 | 0.00 | 0.01 | 0.01 |
| Fat | 0.87 | 0.03 | 0.09 | 0.00 | 0.08 | 0.02 | 0.03 | 0.02 | 0.03 | 0.01 | 0.96 | 0.09 | 0.36 | 0.12 | 0.23 | 0.06 | 0.11 | 0.04 | 0.01 | 0.01 |
| Muscle | 0.19 | 0.09 | 0.01 | 0.00 | 0.01 | 0.00 | 0.01 | 0.00 | 0.02 | 0.01 | 0.16 | 0.04 | 0.02 | 0.01 | 0.01 | 0.01 | 0.01 | 0.00 | 0.00 | 0.00 |
| Bone | 0.12 | 0.03 | 0.02 | 0.01 | 0.02 | 0.00 | 0.02 | 0.02 | 0.14 | 0.02 | 0.20 | 0.12 | 0.06 | 0.02 | 0.04 | 0.02 | 0.03 | 0.01 | 0.00 | 0.00 |
| SG | 0.69 | 0.04 | 0.04 | 0.00 | 0.04 | 0.01 | 0.03 | 0.00 | 0.03 | 0.00 | 0.90 | 0.15 | 0.08 | 0.03 | 0.06 | 0.02 | 0.03 | 0.00 | 0.01 | 0.00 |
| Tail | 1.19 | 0.10 | 0.06 | 0.01 | 0.09 | 0.03 | 0.04 | 0.01 | 0.03 | 0.00 | 0.97 | 0.11 | 0.14 | 0.02 | 0.12 | 0.04 | 0.06 | 0.01 | 0.03 | 0.01 |

**Dosimetric calculations using OLINDA/EXM**

OLINDA/EXM 1.0 was used running under Windows 10, Java 8. Stabin et al, OLINDA/EXM: the second-generation personal computer software for internal dose assessment in nuclear medicine. J Nucl Med. 2005 Jun;46(6):1023-1027

**Supplemental Table 3.** Dosimetry data of [^177^Lu]Lu-rhPSMA-10.1 (1 h voiding interval) using the software OLINDA/EXM 1.0.

| Target Organ | Alpha | Beta | Photon | Total | EDE Cont. | ED Cont. |
| --- | --- | --- | --- | --- | --- | --- |
| Adrenals | 0.00E+00 | 4.61E-04 | 6.17E-04 | 1.08E-03 | 0.00E+00 | 2.69E-06 |
| Brain | 0.00E+00 | 4.61E-04 | 3.86E-05 | 4.99E-04 | 0.00E+00 | 1.25E-06 |
| Breasts | 0.00E+00 | 4.61E-04 | 7.93E-05 | 5.40E-04 | 8.10E-05 | 2.70E-05 |
| Gallbladder Wall | 0.00E+00 | 4.61E-04 | 4.66E-04 | 9.27E-04 | 0.00E+00 | 0.00E+00 |
| LLI Wall | 0.00E+00 | 4.61E-04 | 4.19E-04 | 8.79E-04 | 0.00E+00 | 1.06E-04 |
| Small Intestine | 0.00E+00 | 4.61E-04 | 3.28E-04 | 7.89E-04 | 0.00E+00 | 1.97E-06 |
| Stomach Wall | 0.00E+00 | 4.61E-04 | 2.98E-04 | 7.58E-04 | 0.00E+00 | 9.10E-05 |
| ULI Wall | 0.00E+00 | 4.61E-04 | 3.06E-04 | 7.67E-04 | 0.00E+00 | 1.92E-06 |
| Heart Wall | 0.00E+00 | 2.15E-03 | 2.13E-04 | 2.36E-03 | 1.42E-04 | 0.00E+00 |
| Kidneys | 0.00E+00 | 1.70E-01 | 3.18E-03 | 1.74E-01 | 1.04E-02 | 4.34E-03 |
| Liver | 0.00E+00 | 7.56E-03 | 5.55E-04 | 8.11E-03 | 4.87E-04 | 4.06E-04 |
| Lungs | 0.00E+00 | 8.49E-03 | 2.33E-04 | 8.72E-03 | 1.05E-03 | 1.05E-03 |
| Muscle | 0.00E+00 | 4.61E-04 | 2.00E-04 | 6.61E-04 | 0.00E+00 | 1.65E-06 |
| Ovaries | 0.00E+00 | 4.61E-04 | 4.04E-04 | 8.65E-04 | 2.16E-04 | 1.73E-04 |
| Pancreas | 0.00E+00 | 4.61E-04 | 5.24E-04 | 9.85E-04 | 0.00E+00 | 2.46E-06 |
| Red Marrow | 0.00E+00 | 3.41E-04 | 2.22E-04 | 5.63E-04 | 6.75E-05 | 6.75E-05 |
| Osteogenic Cells | 0.00E+00 | 1.48E-03 | 2.63E-04 | 1.74E-03 | 5.23E-05 | 1.74E-05 |
| Skin | 0.00E+00 | 4.61E-04 | 8.35E-05 | 5.44E-04 | 0.00E+00 | 5.44E-06 |
| Spleen | 0.00E+00 | 2.97E-02 | 1.06E-03 | 3.07E-02 | 1.84E-03 | 7.69E-05 |
| Testes | 0.00E+00 | 4.61E-04 | 2.54E-04 | 7.15E-04 | 0.00E+00 | 0.00E+00 |
| Thymus | 0.00E+00 | 4.61E-04 | 1.00E-04 | 5.61E-04 | 0.00E+00 | 1.40E-06 |
| Thyroid | 0.00E+00 | 4.61E-04 | 5.99E-05 | 5.21E-04 | 1.56E-05 | 2.60E-05 |
| Urinary Bladder Wall | 0.00E+00 | 1.13E-01 | 3.05E-03 | 1.16E-01 | 6.97E-03 | 5.81E-03 |
| Uterus | 0.00E+00 | 4.61E-04 | 7.94E-04 | 1.26E-03 | 0.00E+00 | 3.14E-06 |
| Total Body | 0.00E+00 | 1.62E-03 | 2.20E-04 | 1.84E-03 | 0.00E+00 | 0.00E+00 |

**Effective Dose (mSv/MBq) 1.22E-02**

**Supplemental Table 4.** Dosimetry data of [^177^Lu]Lu-rhPSMA-10.1 (3.5 h voiding interval) using the software OLINDA/EXM 1.0.

| Target Organ | Alpha | Beta | Photon | Total | EDE Cont. | ED Cont. |
| --- | --- | --- | --- | --- | --- | --- |
| Adrenals | 0.00E+00 | 4.61E-04 | 6.35E-04 | 1.10E-03 | 0.00E+00 | 5.48E-06 |
| Brain | 0.00E+00 | 4.61E-04 | 3.86E-05 | 4.99E-04 | 0.00E+00 | 2.50E-06 |
| Breasts | 0.00E+00 | 4.61E-04 | 8.22E-05 | 5.43E-04 | 8.14E-05 | 2.71E-05 |
| Gallbladder Wall | 0.00E+00 | 4.61E-04 | 5.33E-04 | 9.94E-04 | 0.00E+00 | 0.00E+00 |
| LLI Wall | 0.00E+00 | 4.61E-04 | 1.50E-03 | 1.96E-03 | 0.00E+00 | 2.35E-04 |
| Small Intestine | 0.00E+00 | 4.61E-04 | 7.24E-04 | 1.18E-03 | 0.00E+00 | 5.92E-06 |
| Stomach Wall | 0.00E+00 | 4.61E-04 | 3.39E-04 | 7.99E-04 | 0.00E+00 | 9.59E-05 |
| ULI Wall | 0.00E+00 | 4.61E-04 | 5.96E-04 | 1.06E-03 | 0.00E+00 | 5.28E-06 |
| Heart Wall | 0.00E+00 | 2.15E-03 | 2.18E-04 | 2.37E-03 | 0.00E+00 | 0.00E+00 |
| Kidneys | 0.00E+00 | 1.70E-01 | 3.22E-03 | 1.74E-01 | 1.04E-02 | 8.68E-04 |
| Liver | 0.00E+00 | 7.56E-03 | 5.79E-04 | 8.13E-03 | 4.88E-04 | 4.07E-04 |
| Lungs | 0.00E+00 | 8.49E-03 | 2.35E-04 | 8.72E-03 | 1.05E-03 | 1.05E-03 |
| Muscle | 0.00E+00 | 4.61E-04 | 4.42E-04 | 9.02E-04 | 0.00E+00 | 4.51E-06 |
| Ovaries | 0.00E+00 | 4.61E-04 | 1.38E-03 | 1.84E-03 | 4.61E-04 | 3.69E-04 |
| Pancreas | 0.00E+00 | 4.61E-04 | 5.52E-04 | 1.01E-03 | 0.00E+00 | 5.06E-06 |
| Red Marrow | 0.00E+00 | 3.41E-04 | 3.69E-04 | 7.10E-04 | 8.53E-05 | 8.53E-05 |
| Osteogenic Cells | 0.00E+00 | 1.48E-03 | 4.31E-04 | 1.91E-03 | 5.73E-05 | 1.91E-05 |
| Skin | 0.00E+00 | 4.61E-04 | 1.58E-04 | 6.19E-04 | 0.00E+00 | 6.19E-06 |
| Spleen | 0.00E+00 | 2.97E-02 | 1.07E-03 | 3.08E-02 | 1.85E-03 | 1.54E-04 |
| Testes | 0.00E+00 | 4.61E-04 | 9.60E-04 | 1.42E-03 | 0.00E+00 | 0.00E+00 |
| Thymus | 0.00E+00 | 4.61E-04 | 1.02E-04 | 5.63E-04 | 0.00E+00 | 2.82E-06 |
| Thyroid | 0.00E+00 | 4.61E-04 | 6.01E-05 | 5.21E-04 | 1.56E-05 | 2.60E-05 |
| Urinary Bladder Wall | 0.00E+00 | 4.89E-01 | 1.30E-02 | 5.02E-01 | 3.01E-02 | 2.51E-02 |
| Uterus | 0.00E+00 | 4.61E-04 | 3.10E-03 | 3.56E-03 | 2.13E-04 | 1.78E-05 |
| Total Body | 0.00E000 | 1.86E-03 | 4.30E-04 | 2.29E-03 | 0 | 0 |

**Effective Dose (mSv/MBq) 2.85E-02**

**Supplemental Table 5.** Dosimetry data of [^177^Lu]Lu-rhPSMA-10.2 (1 h voiding interval) using the software OLINDA/EXM 1.0.

| Target Organ | Alpha | Beta | Photon | Total | EDE Cont. | ED Cont. |
| --- | --- | --- | --- | --- | --- | --- |
| Adrenals | 0.00E+00 | 2.76E-03 | 1.32E-03 | 4.09E-03 | 2.45E-04 | 1.02E-05 |
| Brain | 0.00E+00 | 2.76E-03 | 2.25E-04 | 2.99E-03 | 0.00E+00 | 7.47E-06 |
| Breasts | 0.00E+00 | 2.76E-03 | 2.22E-04 | 2.99E-03 | 4.48E-04 | 1.49E-04 |
| Gallbladder Wall | 0.00E+00 | 2.76E-03 | 1.03E-03 | 3.80E-03 | 0.00E+00 | 0.00E+00 |
| LLI Wall | 0.00E+00 | 2.76E-03 | 7.11E-04 | 3.48E-03 | 0.00E+00 | 4.17E-04 |
| Small Intestine | 0.00E+00 | 2.76E-03 | 7.42E-04 | 3.51E-03 | 0.00E+00 | 8.77E-06 |
| Stomach Wall | 0.00E+00 | 2.76E-03 | 7.16E-04 | 3.48E-03 | 0.00E+00 | 4.18E-04 |
| ULI Wall | 0.00E+00 | 2.76E-03 | 7.17E-04 | 3.48E-03 | 0.00E+00 | 8.70E-06 |
| Heart Wall | 0.00E+00 | 1.34E-03 | 4.66E-04 | 1.81E-03 | 0.00E+00 | 0.00E+00 |
| Kidneys | 0.00E+00 | 3.32E-01 | 6.30E-03 | 3.38E-01 | 2.03E-02 | 8.46E-03 |
| Liver | 0.00E+00 | 1.27E-02 | 1.08E-03 | 1.37E-02 | 8.25E-04 | 6.87E-04 |
| Lungs | 0.00E+00 | 5.94E-03 | 4.43E-04 | 6.38E-03 | 7.66E-04 | 7.66E-04 |
| Muscle | 0.00E+00 | 2.76E-03 | 4.55E-04 | 3.22E-03 | 0.00E+00 | 8.05E-06 |
| Ovaries | 0.00E+00 | 2.76E-03 | 7.25E-04 | 3.49E-03 | 8.72E-04 | 6.98E-04 |
| Pancreas | 0.00E+00 | 2.76E-03 | 1.14E-03 | 3.91E-03 | 0.00E+00 | 9.77E-06 |
| Red Marrow | 0.00E+00 | 2.05E-03 | 5.42E-04 | 2.59E-03 | 3.11E-04 | 3.11E-04 |
| Osteogenic Cells | 0.00E+00 | 8.88E-03 | 7.65E-04 | 9.65E-03 | 2.89E-04 | 9.65E-05 |
| Skin | 0.00E+00 | 2.76E-03 | 2.29E-04 | 2.99E-03 | 0.00E+00 | 2.99E-05 |
| Spleen | 0.00E+00 | 4.27E-02 | 1.89E-03 | 4.46E-02 | 2.67E-03 | 1.11E-04 |
| Testes | 0.00E+00 | 2.76E-03 | 4.39E-04 | 3.20E-03 | 0.00E+00 | 0.00E+00 |
| Thymus | 0.00E+00 | 2.76E-03 | 3.23E-04 | 3.09E-03 | 0.00E+00 | 7.72E-06 |
| Thyroid | 0.00E+00 | 2.76E-03 | 2.94E-04 | 3.06E-03 | 9.17E-05 | 1.53E-04 |
| Urinary Bladder Wall | 0.00E+00 | 1.06E-01 | 3.05E-03 | 1.09E-01 | 6.52E-03 | 5.43E-03 |
| Uterus | 0.00E+00 | 2.76E-03 | 1.08E-03 | 3.84E-03 | 0.00E+00 | 9.60E-06 |
| Total Body | 0 | 4.7e-3 | 5.06e-4 | 5.21E-03 | 0 | 0 |

**Effective Dose (mSv/MBq) 1.78E-02**

**Supplemental Table 6.** Dosimetry data of [^177^Lu]Lu-rhPSMA-10.2 (3.5 h voiding interval) using the software OLINDA/EXM 1.0.

| Target Organ | Alpha | Beta | Photon | Total | EDE Cont. | ED Cont. |
| --- | --- | --- | --- | --- | --- | --- |
| Adrenals | 0.00E+00 | 2.76E-03 | 1.34E-03 | 4.10E-03 | 0.00E+00 | 2.05E-05 |
| Brain | 0.00E+00 | 2.76E-03 | 2.26E-04 | 2.99E-03 | 0.00E+00 | 1.49E-05 |
| Breasts | 0.00E+00 | 2.76E-03 | 2.25E-04 | 2.99E-03 | 4.48E-04 | 1.49E-04 |
| Gallbladder Wall | 0.00E+00 | 2.76E-03 | 1.08E-03 | 3.85E-03 | 0.00E+00 | 0.00E+00 |
| LLI Wall | 0.00E+00 | 2.76E-03 | 1.55E-03 | 4.31E-03 | 0.00E+00 | 5.17E-04 |
| Small Intestine | 0.00E+00 | 2.76E-03 | 1.05E-03 | 3.81E-03 | 0.00E+00 | 1.91E-05 |
| Stomach Wall | 0.00E+00 | 2.76E-03 | 7.48E-04 | 3.51E-03 | 0.00E+00 | 4.21E-04 |
| ULI Wall | 0.00E+00 | 2.76E-03 | 9.41E-04 | 3.71E-03 | 0.00E+00 | 1.85E-05 |
| Heart Wall | 0.00E+00 | 1.34E-03 | 4.69E-04 | 1.81E-03 | 0.00E+00 | 0.00E+00 |
| Kidneys | 0.00E+00 | 3.32E-01 | 6.33E-03 | 3.39E-01 | 2.03E-02 | 1.69E-03 |
| Liver | 0.00E+00 | 1.27E-02 | 1.09E-03 | 1.38E-02 | 8.26E-04 | 6.88E-04 |
| Lungs | 0.00E+00 | 5.94E-03 | 4.44E-04 | 6.39E-03 | 7.66E-04 | 7.66E-04 |
| Muscle | 0.00E+00 | 2.76E-03 | 6.42E-04 | 3.41E-03 | 0.00E+00 | 1.70E-05 |
| Ovaries | 0.00E+00 | 2.76E-03 | 1.48E-03 | 4.25E-03 | 1.06E-03 | 8.50E-04 |
| Pancreas | 0.00E+00 | 2.76E-03 | 1.16E-03 | 3.93E-03 | 0.00E+00 | 1.96E-05 |
| Red Marrow | 0.00E+00 | 2.05E-03 | 6.56E-04 | 2.70E-03 | 3.24E-04 | 3.24E-04 |
| Osteogenic Cells | 0.00E+00 | 8.88E-03 | 8.95E-04 | 9.78E-03 | 2.93E-04 | 9.78E-05 |
| Skin | 0.00E+00 | 2.76E-03 | 2.87E-04 | 3.05E-03 | 0.00E+00 | 3.05E-05 |
| Spleen | 0.00E+00 | 4.27E-02 | 1.90E-03 | 4.46E-02 | 2.67E-03 | 2.23E-04 |
| Testes | 0.00E+00 | 2.76E-03 | 9.85E-04 | 3.75E-03 | 0.00E+00 | 0.00E+00 |
| Thymus | 0.00E+00 | 2.76E-03 | 3.24E-04 | 3.09E-03 | 0.00E+00 | 1.54E-05 |
| Thyroid | 0.00E+00 | 2.76E-03 | 2.94E-04 | 3.06E-03 | 9.17E-05 | 1.53E-04 |
| Urinary Bladder Wall | 0.00E+00 | 3.97E-01 | 1.08E-02 | 4.08E-01 | 2.45E-02 | 2.04E-02 |
| Uterus | 0.00E+00 | 2.76E-03 | 2.86E-03 | 5.62E-03 | 3.37E-04 | 2.81E-05 |
| Total Body | 0 | 4.89e-3 | 6.69e-4 | 5.56e-2 | 0 | 0 |

**Effective Dose (mSv/MBq) 2.64E-02**


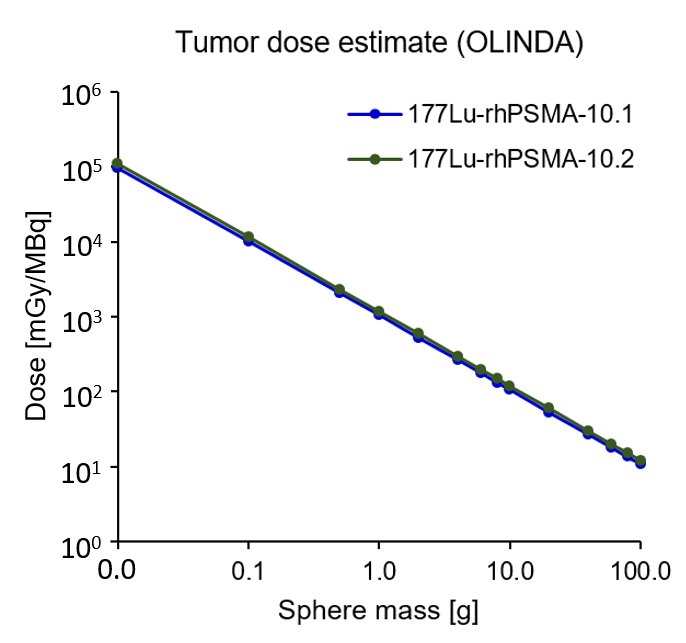


**Supplemental Figure 3.** Comparative tumor dose estimate of [^177^Lu]Lu-rhPSMA-10.1 and [^177^Lu]Lu-rhPSMA-10.2 using software OLINDA/EXM 1.0.

**Supplemental Table 7.** Biodistribution studies of [^177^Lu]Lu-rhPSMA-10.1 and [^177^Lu]Lu-rhPSMA-10.2 in mice bearing LNCaP tumors at 1, 24 and 168 h p.i. Values are expresses as a percentage of injected dose per gram (%ID/g), mean ± SD (n = 4-5). SI: small intestine; LI: large intestines.

| Radioligand | [^177^Lu]Lu-rhPSMA-10.1 | | | | | | | |  | [^177^Lu]Lu-rhPSMA-10.2 | | | | | | | | |
| --- | --- | --- | --- | --- | --- | --- | --- | --- | --- | --- | --- | --- | --- | --- | --- | --- | --- | --- |
| Time point | 1 h | |  | 24 h | |  | 168 h | |  | 1 h | |  | 24 h | |  | 168 h | |  |
|  | Mean %ID/g | ±SD |  | Mean %ID/g | ±SD |  | Mean %ID/g | ±SD |  | Mean %ID/g | ±SD |  | Mean %ID/g | ±SD |  | Mean %ID/g | ±SD |  |
| Blood | 0.59 | 0.15 |  | 0.00 | 0.00 |  | 0.00 | 0.00 |  | 0.34 | 0.06 |  | 0.01 | 0.00 |  | 0.00 | 0.00 |  |
| Heart | 0.28 | 0.05 |  | 0.02 | 0.01 |  | 0.01 | 0.00 |  | 0.24 | 0.03 |  | 0.02 | 0.00 |  | 0.01 | 0.00 |  |
| Lung | 1.20 | 0.27 |  | 0.05 | 0.02 |  | 0.06 | 0.08 |  | 1.38 | 0.34 |  | 0.05 | 0.01 |  | 0.02 | 0.00 |  |
| Liver | 0.44 | 0.16 |  | 0.24 | 0.13 |  | 0.13 | 0.11 |  | 0.45 | 0.20 |  | 0.23 | 0.18 |  | 0.07 | 0.02 |  |
| Spleen | 21.45 | 7.61 |  | 0.25 | 0.10 |  | 0.11 | 0.04 |  | 37.69 | 11.22 |  | 0.33 | 0.12 |  | 0.16 | 0.11 |  |
| Pancreas | 0.42 | 0.08 |  | 0.03 | 0.02 |  | 0.01 | 0.00 |  | 0.49 | 0.07 |  | 0.02 | 0.00 |  | 0.01 | 0.00 |  |
| Kidneys | 170.34 | 34.43 |  | 2.80 | 1.13 |  | 0.17 | 0.04 |  | 223.91 | 23.42 |  | 14.86 | 6.60 |  | 0.33 | 0.11 |  |
| Stomach | 0.69 | 0.73 |  | 0.05 | 0.02 |  | 0.02 | 0.02 |  | 0.35 | 0.07 |  | 0.04 | 0.01 |  | 0.01 | 0.00 |  |
| SI | 0.34 | 0.19 |  | 0.04 | 0.01 |  | 0.01 | 0.00 |  | 0.26 | 0.05 |  | 0.04 | 0.02 |  | 0.02 | 0.01 |  |
| LI | 0.41 | 0.20 |  | 0.06 | 0.02 |  | 0.02 | 0.01 |  | 0.20 | 0.01 |  | 0.08 | 0.04 |  | 0.04 | 0.01 |  |
| Muscle | 0.21 | 0.08 |  | 0.01 | 0.00 |  | 0.00 | 0.00 |  | 0.18 | 0.01 |  | 0.01 | 0.00 |  | 0.00 | 0.00 |  |
| Bone | 0.18 | 0.09 |  | 0.02 | 0.02 |  | 0.01 | 0.01 |  | 0.10 | 0.06 |  | 0.01 | 0.01 |  | 0.00 | 0.00 |  |
| Salivary gland | 0.94 | 0.28 |  | 0.05 | 0.01 |  | 0.02 | 0.01 |  | 1.54 | 0.29 |  | 0.06 | 0.02 |  | 0.02 | 0.01 |  |
| Tail | 0.95 | 0.26 |  | 0.06 | 0.02 |  | 0.03 | 0.02 |  | 0.88 | 0.14 |  | 0.06 | 0.02 |  | 0.03 | 0.01 |  |
| Tumor | 8.58 | 1.65 |  | 11.39 | 7.67 |  | 2.21 | 0.81 |  | 10.93 | 1.95 |  | 8.89 | 2.00 |  | 4.12 | 1.41 |  |
